# Effect of surrounding landscape on *Popillia japonica* abundance and their spatial pattern within Wisconsin vineyards

**DOI:** 10.3389/finsc.2022.961437

**Published:** 2022-10-27

**Authors:** Jacob Henden, Christelle Guédot

**Affiliations:** Department of Entomology, University of Wisconsin-Madison, Madison, WI, United States

**Keywords:** Japanese beetle, invasive, landscape effect, grape, EIQ, longitude, abiotic factors

## Abstract

Landscapes surrounding agroecosystems can provide resources that may benefit insect pests. This project examined the influence of the surrounding landscape on the abundance and spatial pattern of *Popillia japonica* (Coleoptera: Scarabaeidae) in vineyards. Twenty vineyards across Southern Wisconsin, spanning a gradient of 5-80% cropland in a 1.5km radius surrounding landscape, were sampled in 2017 and 2018 for *P. japonica* adults and leaf injury. The distribution of *P. japonica* and leaf injury was assessed by sampling along a transect at the edge, halfway from the edge to the center, and at the center of each vineyard. The proportion of cropland and pasture in the surrounding landscape along with abiotic factors of temperature, precipitation, longitude, and pesticide use (determined using Environmental Impact Quotient) were included in models to explain the variation of *P. japonica* abundance and leaf injury. No significant relationship was observed between proportion cropland in the surrounding landscape and *P. japonica* abundance or leaf injury. Combined effects of pasture, longitude, and temperature best explained variation in the abundance of *P. japonica* adults while longitude, temperature and EIQ best explained variability in leaf injury. Vineyards with more pastures in the surrounding landscape, located further east, and with higher temperatures, generally had more *P. japonica* adults and vineyards further east with higher temperature and lower EIQ pesticide use generally had higher levels of leaf injury. Additionally, variability in weekly temperature and precipitation influenced weekly abundance, with higher temperatures and less precipitation resulting in greater weekly abundance of *P. japonica* adults. Significantly more adult *P. japonica* and greater leaf injury were found at the edges than in the center of vineyards. Our results suggest beetles from the surrounding landscape likely contribute to populations of *P. japonica* adults found feeding on vines on vineyard edges, and *P. japonica* abundance and associated leaf injury are influenced by geographical location, local weather conditions, and pesticide use.

## Highlights

• The amount of pasture in the surrounding landscape, longitude and temperature best explained the abundance of *P. japonica* adults
• Vineyards further east with higher temperature and lower EIQ pesticide use experienced higher levels of leaf injury.
• Higher *P. japonica* populations and greater leaf injury were found at vineyard edges
• Management of *P. japonica* populations could be targeted towards vineyard edges

## Introduction

The surrounding landscape can influence the abundance of pest insects within agricultural fields by providing shelter, refuge, and nutritional resources throughout the year (1, 2), by acting as natural barriers to movement (3), or by supporting populations of natural enemies which help suppress pest populations (4, 5). Insect diversity generally increases with a higher proportion of natural or uncultivated land in the surrounding landscape (6–8), but many insect pests may benefit from a higher proportion of cultivated land surrounding target crops (9, 10). Highly mobile and polyphagous insect pests can utilize a variety of alternative host plants across large areas and can be influenced by the availability of resources in surrounding cultivated and uncultivated land (11, 12). Understanding how the composition of landscape surrounding agroecosystems affects pest populations can be critical in assessing risk and implementing management strategies for particular pests.

*Popillia japonica* (Newman) (Coleoptera: Scarabaeidae), a widely established invasive herbivore across the Eastern and Central U.S (13). known to feed on over 300 plant species, has become the key pest of several small fruits, ornamentals, and field crops (14, 15). The larvae of *P. japonica* cause damage to grasses by feeding on roots and the adults through defoliation of leaves and sometimes direct feeding on fruits (16). The adult beetles have a strong flight capacity, being able to sustain flight for over 5 km (17), which allows them to move between habitats and feed on numerous host plant species including wild and cultivated species (16). Additionally, in the U.S. *P. japonica* does not appear to experience pressure from natural enemies that would significantly influence their populations (13). A previous study suggested that the density of *P. japonica* populations is largely determined by the total availability of preferred host plants in the area, and that emerging adults fly to and aggregate at sites with an abundance of preferred host plants (18). Landscapes with higher proportions of cropland in the surrounding area have previously been shown to result in higher number of adults captured in traps in Illinois, likely due to the availability of corn and soybean which are both suitable hosts to *P. japonica* adults (19). In addition to preferred adult host plants, *P. japonica* can also be influenced by the availability of optimal oviposition sites or areas where there is high soil moisture (20, 21) and where there is sunlight and short grass cover (22). These conditions are often found in pastures and turfgrasses, and the presence of these can also increase the prevalence of *P. japonica* (23, 24).

As *P. japonica* are generalists, suitable host plants and oviposition sites may be available within and outside of susceptible agricultural crops. For example, highbush blueberry fields offer suitable oviposition sites between rows of plants where larvae can overwinter, as well as preferred host plants for adults to feed on (14). Larvae and adults were found to be more abundant towards the edges of fields in blueberry (25) and soybean (26), and the number of larvae found in the spring in blueberry fields was correlated to the number of adults found later in the summer which suggests the overwintering larvae significantly contribute to the number of adults found feeding on blueberry plants in the summer (25). Grapes, which have an economic value estimated nationally at over $6 billion per year (27), are a preferred host of adults (28), and turfgrass, which is commonly used as ground cover within vineyards, is a highly suitable oviposition site for these beetles (16). It is unclear what proportion of *P. japonica* adults found feeding on grapevines move in from the surrounding landscape or overwinter as larvae within a vineyard. How different landscape types contribute to populations of *P. japonica* within agroecosystems is important to understanding their dispersal, and for growers to assess risk based on the composition of the landscape surrounding their farm.

Abiotic factors, such as precipitation and temperature, can affect *P. japonica* population abundance and feeding behavior. Higher precipitation can influence *P. japonica* abundance as beetles prefer to oviposit in wetter soils (20, 21), and variability in summer rainfall may explain year to year variability in *P. japonica* abundance as the larvae survive better in wetter conditions (29). Temperature may also influence *P. japonica* behavior as adults tend to have higher consumption rates at higher temperatures (30); however, no research has addressed the impact temperature and precipitation may have on adult abundance in vineyards.

The main objectives of this study were to assess how the surrounding landscape along with other abiotic factors influence *P. japonica* populations and to determine the distribution of adults from field edges to field interiors in vineyards. To address these objectives, we conducted a two-year, season-long study assessing *P. japonica* adult population abundance and leaf injury at selected vineyards across Southern Wisconsin with variable landscape composition surrounding vineyards. We expected to see a higher abundance of *P. japonica* and associated leaf injury as the amount of cropland increased in the landscape surrounding vineyards and higher population densities as well as increased leaf injury near field margins compared to field interiors in vineyards. A better understanding of how the surrounding landscape and other abiotic factors contribute to the abundance of *P. japonica* and how beetles are distributed within vineyards can provide valuable information for assessing risk and can allow growers to make more targeted applications of pesticides (31) to manage this detrimental pest.

## Materials and methods

To assess the effect of the proportion of cropland in the surrounding landscape on *P. japonica* abundance in vineyards, we conducted a two-year study from June to September in 2017 and 2018, when *P. japonica* are known to occur in Southern Wisconsin (32). We monitored *P. japonica* adults as well as leaf injury caused by adults at 20 vineyards across Southern Wisconsin, USA (**Supplemental Table 1**). The average vineyard size was approximately 12000 m^2^, with a range of 4000-32000 m^2^, and the average transect length was 87 m, with a range of 50-150 m. Vineyards were all planted with a mixed variety of cold climate grapes.

### Site selection

The surrounding landscape of vineyards were evaluated and described using satellite-derived Cropland Data Layer (NASS USDA 2017) using ArcMap 10.3 (ESRI, Redlands, CA). The composition of the surrounding landscape was calculated around a 1.5 km radius from the center of each vineyard based on typical dispersal of *P. japonica* shown in capture-mark-recapture experiments (17). Data from landcover classes was reclassified to focus on broader landscape categories of interest (33, 34): 1) Cropland, which included all cultivated crops excluding hay and non-alfalfa pastures; 2) Woodland, which included forests, deciduous forests, mixed forests and woody wetlands; 3) Non-pasture Grassland, which included non-alfalfa hay, shrublands, herbaceous wetlands, and fallow croplands, and; 4) Pasture, which included only the class specified as “Grassland/Pasture” in the Cropland Data Layer (NASS USDA 2017) (**Supplemental Table 2**).

**Figure 1:**
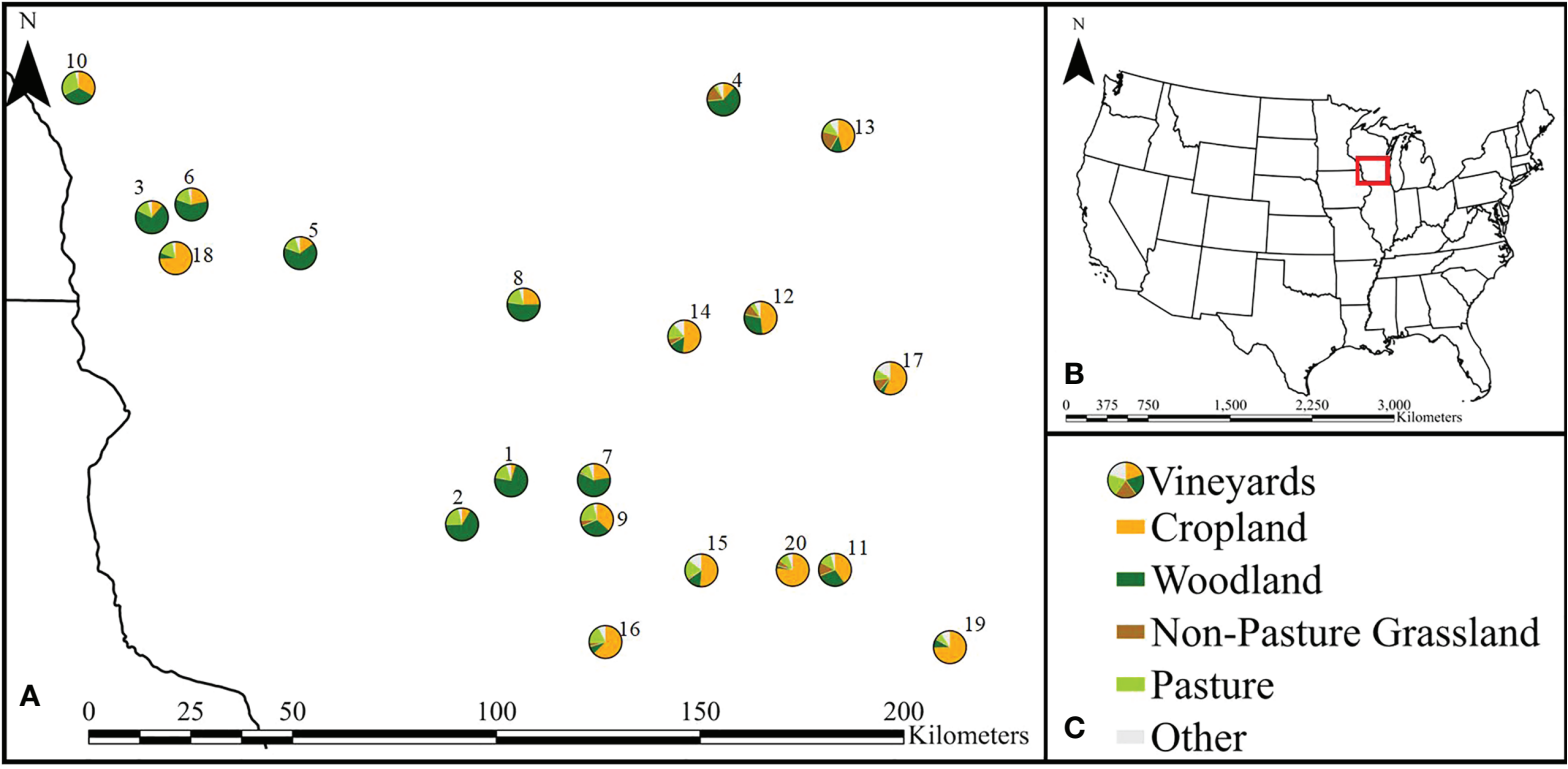
**(A)** Map showing the location of the 20 vineyards with pie charts of the composition of the respective 1.5 km surrounding landscapes for each vineyard across Southern Wisconsin where sampling occurred. Vineyards are labeled 1-20 and correspond to those listed in **Supplemental Table 1**. **(B)** Locator map to give spatial reference to map shown in panel **(A)**. **(C)** Legend indicating different landscape types (detailed in **Supplemental Table 1**).

In Southern Wisconsin, grassland and pastures were relatively uncommon land cover types surrounding potential vineyards we would sample at, while cropland and woodland were more prevalent landcover types. We selected 20 vineyards with less than 30% pasture and non-pasture grassland in the surrounding landscape and where at least 75% of the surrounding landscape was comprised of non-pasture grassland, pasture, cropland, and woodland (**Figure 1**). Selected vineyards spanned a gradient from low to high (4.8-78%) cropland in the surrounding landscape (**Supplemental Table 1**).

### Experimental design

**Figure 2:**
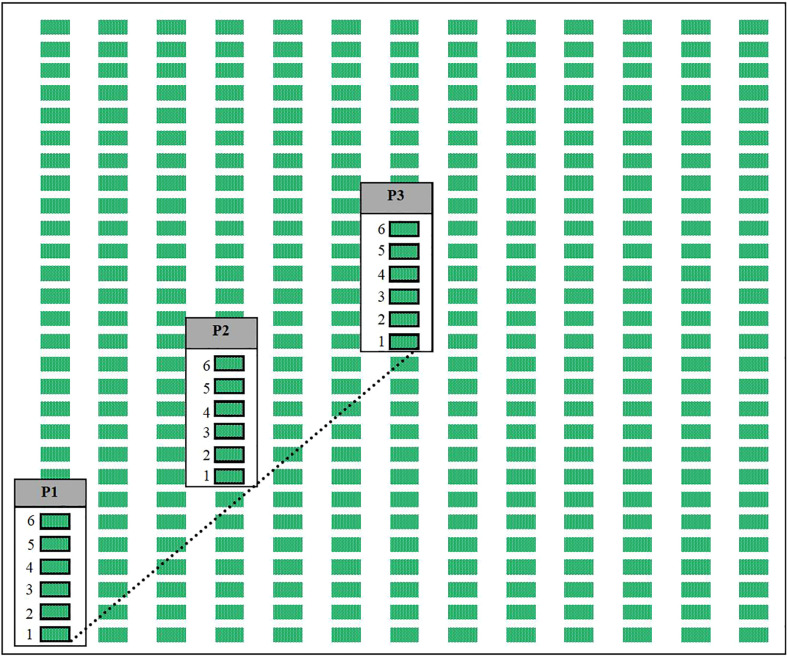
Simplified diagram of a vineyard field to show our experimental design. At each vineyard a diagonal transect (represented by the dashed line) was established from the corner of a field to its center, and we would sample from grape plants within the rows at three points (labeled as P1, P2, and P3) representing the edge of the field, a point halfway from the edge to center, and the center of the field respectively. Each week at every sampling point adult *P. japonica* would be collected from 6 vines (shown as rectangles outlined in black, labeled 1-6), and one vine per sampling point would be used for leaf injury assessment starting with the first vine (labeled as 1) on the first week, to the second vine (labeled as 2) on the second week, until the seventh week where we would start again at the first vine (labeled as 1) and cycle through again.

To assess the distribution from field edges to field interiors of *P. japonica* within vineyards, we set up a diagonal linear transect at each vineyard, which consisted of three sampling locations: one at the edge of the vineyard, one at the halfway point from the edge to the center, and one at the center of the vineyard (**Figure 2**). For each diagonal transect, the sampling location at the edge was established at a corner of the largest and most rectangular available field within the vineyard. Corners were selected which would allow for the maximum possible length of the diagonal transect. When multiple corners fit these criteria, we selected a corner that had at least two different types of bordering landscapes to reduce the impact of a single bordering landscape on the sampling design. Each vineyard was sampled for adult *P. japonica* and estimates of leaf damage were taken once a week between the hours of 09:00 and 17:00. Sampling began in mid-June, before the start of adult *P. japonica* emergence (32) and continued until no more adults were observed at the vineyards for two consecutive weeks, resulting in 17 weeks of sampling in 2017 and 16 weeks of sampling in 2018.

### Adult sampling and leaf injury assessment

To determine the abundance of *P. japonica* adults in vineyards, we carefully hand-collected all adults present on sections of the 6 grapevines associated with each of the sampling locations, the first vine (out of 6 plants sampled at each sampling point) intersects with the established transect and the next 5 grape plants were located sequentially along the row of plants away from the edge of the vineyard where the transect begins (**Figure 2**). Collected adults were placed into a container with soapy water and were then placed in a freezer at -20°C upon return to the laboratory where they were later counted.

Each week, leaf injury was estimated at one of the six vines at each sampling location and 40 leaves on the closest shoot to the center of the vine were counted starting at the distal end of the shoot. For consistency across all of our counts and to avoid possible variability that may occur in different parts of the plant we always started with leaves on the most central shoot. If a shoot did not have 40 leaves, the next adjacent shoot was counted until 40 leaves were assessed. Each week, vines sampled were alternated as we would sample the first vine (out of the 6 per sampling point) on the first week of sampling, and then the second vine on the second week of sampling, until the seventh week where we would start again at the first vine and cycle through the vines again, additionally the shoots sampled were alternated (left or right of the center of the vine every other week). Leaf injury assessment was conducted following methods by Boucher and Pfeiffer (35) by counting the number of leaves visually estimated to have at least 10% area loss due to *P. japonica* feeding damage characterized by the skeletonization of leaves. Leaf counts and associated leaf injury estimates were removed from our dataset for the 3^rd^ week of sampling in 2017 as the methodology used for leaf counts during this week was not consistent with our other weekly assessments.

### Weather conditions

To assess the impact that temperature and precipitation may have on the abundance of *P. japonica* adults throughout the season, estimates of weather conditions at each vineyard throughout the course of adult emergence were obtained from Oregon State University Parameter-elevation Regressions on Independent Slopes (PRISM) Model (http://prism.oregonstate.edu). The spatial resolution for this weather data was 4km. The average daily temperature and precipitation for vineyards from June to September in 2017 and 2018 were downloaded to evaluate conditions when adults were present.

### Pesticide usage

To account for variable levels of insecticide applications between vineyards used to manage *P. japonica*, an Environmental Impact Quotient (EIQ) was calculated using an online field use EIQ calculator from Cornell University (nysipm.cornell.edu/eiq/calculator-field-use-eiq/) for each vineyard and each year of the study. The EIQ uses the rate and frequency of pesticide applications combined with toxicological and environmental data available on those pesticides to generate a standardized score that can be used to compare management programs (36). For our purposes, the average EIQ per year for each vineyard was calculated based only on insecticide applications and did not account for fungicide or herbicide applications. We produced yearly EIQ estimates per vineyard as each vineyard sprayed insecticides on their own schedule not allowing for weekly EIQ estimates across vineyards. No sampling occurred immediately following a pesticide application. The date, active ingredient, and application rate of all insecticides was self-reported by each vineyard manager for each year and the average EIQ values across the two years of the study for the different vineyards ranged from 0-140.

### Vineyard location

We included longitude values for each vineyard in our data analysis, to account for the potential variations in levels of *P. japonica* establishment across Eastern to Western Wisconsin. As our vineyards were spread throughout Southern Wisconsin, they span different broader ecological landscapes with variable climate, soils, hydrology, and landcover (37), which could influence the establishment of *P. japonica* populations (17, 38).

### Data analysis

Multiple regressions were used to build two sets of models to explain the effect of cropland, pasture, precipitation and temperature throughout the season (average of both years from June to September), the average EIQ of the vineyard (averaged across both years), and the longitude of the vineyard on the abundance of *P. japonica* adults (the average number of adult *P. Japonica* collected per vineyard per week across the two years) and estimated proportion of leaf injury (average estimated proportion of leaf injury recorded per vineyard per week across the two years). Interaction effects between variables were also included in these two sets of models.

Each set of models were evaluated based on Akaike Information Criterion (AIC) with a correction for small sample size (AICc), where models with lower AICc scores were considered to better fit the data (39). Models were also evaluated for multicollinearity, and models where any variable included had a variance inflation factor greater than 10 were excluded (40). Additionally, due to our relatively small sample size (n=20) we chose to include no more than three variables in a given model. A logarithmic transformation was also applied to the abundance of adults in order for the data to better fit assumptions of equal variance and normality (41). In each set of models we included all possible combinations of the independent variables and interaction effects that were not excluded by our previous mentioned criteria (**Supplemental Tables 3** and **4**). We additionally used this same model selection process, but modified the variables of cropland and pasture to reflect their proportion in the landscape surrounding vineyards at different buffer widths (0.5km, 1km, 1.5km, 2.5km, 5km, 10km). However, across different spatial scales there was no change in what individual factors were statistically significant or which models were evaluated to be better based on AICc, and therefore we chose to only present data for the 1.5 km spatial scale

Multiple regressions were also used to build a set of models to explain the effect of vineyard, week of sampling, year, weekly precipitation, and weekly temperature (for temperature and precipitation variables 7 days were averaged, consisting of the 6 days prior to when sampling occurred along with the day of sampling itself), on the weekly average abundance of *P. japonica* adults (**Supplemental Table 5**). A quadratic term for week sampled was also added to better fit the models to the seasonal variation in our data. We included vineyard, week of sampling, and year in all models built also testing possible combinations of precipitation, temperature, and interactions between precipitation and temperature. These models were evaluated based on Akaike Information Criterion (AIC) and models with lower AIC scores were considered to better fit the data. A logarithmic transformation was applied to the abundance of adults (per vineyard per week) in order for the data to better fit assumptions of equal variance and normality (41).

A Pearson’s correlation test was used to assess correlations between the abundance of *P. japonica* adults and estimated proportion of leaf damage. The same test was used to assess the correlation between the proportion of cropland and the proportion of woodland in the surrounding landscape and the correlation between the longitude of the vineyard and the amount of cropland in the surrounding landscape.

One-way ANOVA tests were used to compare the abundance of adults and estimated proportion leaf injury between the three sampling points to determine if there were significant differences between the relative abundance of beetles and estimated leaf injury across different sampling points. Following a significant p-value for the ANOVA, a Tukey HSD test was used to perform pairwise comparisons between sampling points. All statistical analysis was performed in R (R Development Core Team 2021).

## Results

The proportion of cropland and woodland surrounding vineyards were strongly negatively correlated (*r*(18)=-0.98, *p* <.001),indicating that from our selected sites, vineyards surrounded by less cropland generally had more woodland in the surrounding landscape. The proportion of cropland in the surrounding landscape and the longitude of the vineyard were somewhat positively correlated (*r*(18)=0.47, *p*<0.04), as selected vineyards located further east in Wisconsin had on average higher amounts of cropland in the surrounding landscape than vineyards further west.

The best-fitting model (lowest AICc) for the average adult *P. japonica* abundance included pasture, temperature, and longitude (R^2 =^ 0.75, *p*<0.001) (**Table 1**). This model shows adult abundance being higher in vineyards with more pastures in the surrounding landscape, with higher temperatures, and located further east in Southern Wisconsin.

**Table 1:** Parameter estimates ± SE of selected model (R^2^ = 0.75, *p*<0.001) explaining variation in the average abundance of average *P. japonica* adults per vineyard per week (transformed by log base 10).

Coefficients	Parameter Estimate ± SE	t value	Pr>(|t|)
**Intercept**	71.84 ± 18.78	3.83	<0.01
**Pasture**	0.06 ± 0.02	2.38	0.03
**Temp**	1.09 ± 0.36	3.01	<0.01
**Long**	1.03 ± 0.17	5.94	<0.002

Variables included in the selected model were: 1) averaged daily temperature (°C) from June-September of 2017 and 2018 at the vineyard (temp) and 2) longitude value of the center of each vineyard (long).

The best-fitting model for the average estimated proportion of leaf injury included temperature, longitude, and EIQ (R^2^ = 0.44, *p*=0.007) (**Table 2**), with vineyards located further east, with less intensive pesticide use (lower EIQ), and higher temperatures generally having higher levels of leaf injury than vineyards further west, with higher levels of pesticide use, and lower temperatures.

**Table 2:** Parameter estimates ± SE of selected model (R^2^ = 0.44, *p*=0.007) explaining variation in average leaf injury observed at vineyards.

Coefficients	Parameter Estimate ± SE	t value	Pr>(|t|)
**Intercept**	4.14 ± 2.89	1.43	0.17
**Temp**	0.15 ± 0.06	2.36	0.03
**EIQ**	-0.002 ± 0.0001	-2.87	0.01
**Long**	0.08 ± 0.03	2.67	0.02

Variables included in the selected model were: 1) averaged daily temperature (°C) from June-September of 2017 and 2018 at the vineyard (temp); 2) Environmental Impact Quotient score for the vineyard averaged across 2017 and 2018 (EIQ); and 3) longitude value of the center of each vineyard (long).

The best fitting model for weekly *P. japonica* abundance included year, week, vineyard, weekly precipitation, and weekly temperature (R^2^= 0.51, *p*<0.001) (**Table 3**) (**Supplemental Table 6**), with the models indicating that higher temperatures and less precipitation in the week leading up to sampling resulted in a greater weekly abundance of *P. japonica*.

**Table 3:** Analysis of variance table for the selected model explaining variability in weekly abundance of *P. japonica* across 20 vineyards.

	Df	Sum Sq	Mean Sq	F value	Pr (>F)
**Week^2^**	1	0.31	0.31	0.22	0.64
**Week**	1	351.80	351.80	243.80	<0.0001
**Year**	1	3.04	3.04	2.10	0.15
**Vin**	19	559.95	29.47	20.42	<0.0001
**Precip**	1	5.93	5.93	4.11	0.04
**Temp**	1	11.16	11.16	7.74	<0.01
**Residuals**	575	829.72	1.44		

The variables included in models include 1) Week (the week we sampled, 1^st^ week for the year; 2)Year (year of sampling, 2017 or 2018); 3) Vin (vineyard sampled V01-V20); 4) Precip (average precipitation at the vineyard sampled for the previous 7 days); and 5) average temperature at the vineyard sampled for the previous 7 days.

**Figure 3:**
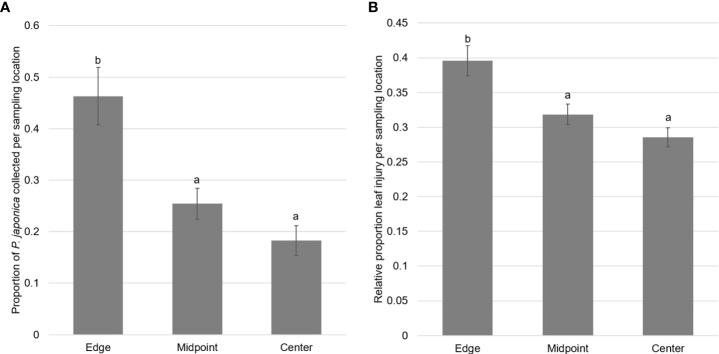
**(A)** Mean proportion ± SEM of adult *P. japonica* collected from three sampling locations along transects from field edges to field interiors at twenty vineyards. Different letters signify significant differences between the means of groups (*p*<0.05). **(B)** Mean relative estimated leaf injury ± SEM from three sampling locations along transects from field edges to field interiors at twenty vineyards. Different letters signify significant differences between the means of groups (*p*<0.05).

The abundance of adults and the estimated proportion of leaf injury were positively correlated (*r*(18)=0.87, *p* <.001) indicating that there was more leaf injury when adult beetles were more abundant. There were significant differences in the proportion of adults collected at the different points along the transects (*F*_2,57_ = 12.44, *p*<0.00001), with a higher proportion of adult *P. japonica* collected at the edges of the vineyards (Mean ± SEM: 0.46 ± 0.06) compared to the center (0.18 ± 0.03; *t*_17_ = 4.17, *p*<0.001) and halfway from the edge to the center (0.25 ± 0.03; *t*_17_ = 3.13, *p*=0.002) (**Figure 3**). Leaf injury varied between sampling locations (*F*_2,57_ = 10.49, *p*<0.0001), with a significantly higher relative amount of estimated leaf injury at the edges of the vineyards (0.40 ± 0.02) compared to both the center (0.29 ± 0.14) (*t*_17_ = 1.23, *p*=0.0001) and halfway from the edge to the center of the vineyards (0.32 ± 0.01) (*t*_17_ = 2.06, *p*=0.008) (**Figure 3**).

## Discussion

Our results showed that the location of the vineyards, the proportion of pastures in the surrounding landscape, and temperature best explained the variability in *P. japonica* adult populations, while location, temperature, and pesticide usage best explained variability in leaf injury. Vineyards located further east with higher temperatures and more pasture in the surrounding landscape had more *P. japonica* adults present and vineyards further east with higher temperatures and lower EIQ pesticide usage had greater levels of leaf injury. We also found that week, year, and vineyard beetles were sampled from along with the prior week’s variability in temperature and precipitation best explained weekly variability in *P. japonica* abundance across vineyards, with a greater abundance of *P. japonica* typically sampled when the temperature of the previous week was higher, and the precipitation was lower. Our results also showed an edge-biased distribution of *P. japonica* adults and leaf injury along our established transects, with more adults and leaf injury near the vineyard edge compared to the interior of the vineyards.

We hypothesized that more *P. japonica* adults would be more abundant as the amount of cropland increased in the landscape. Contrary to our expectation, the amount of cropland in the surrounding landscape, as defined in this study, did not have a significant effect on *P. japonica* abundance or leaf injury. The composition of the surrounding landscape can influence pest populations (2, 7, 8), and we did find landscape, specifically the proportion of pastures in the surrounding landscape, helped explain more variability in *P. japonica* abundance across vineyards. Our findings of greater *P. japonica* abundance at vineyards with more pasture in the surrounding landscape are consistent with previous research and may be driven by the availability of highly suitable oviposition sites for *P. japonica* (23, 24). We had also expected to find an impact from the amount of cultivated land as previous studies suggest that more cultivated land in the surrounding landscape can increase population densities for a variety of insect pests (2, 9, 42). Here, the proportion of cropland in the surrounding landscape was less important than the geographical location, management practices of the vineyard, and proportion of pastures in the surrounding landscape in explaining the variation of *P. japonica* adults and leaf injury. As an invasive species with minimal pressure from natural enemies (13), *P. japonica* may likely not experience the indirect effects of uncultivated landscapes supporting larger populations of natural enemies that may contribute to pest suppression (e.g. 43–45), but would be expected to be influenced by resource availability from cultivated or uncultivated landscapes (42). *Popillia japonica* are extreme generalists and highly mobile (17, 46), which may allow them to utilize a wide variety of resources across landscapes with variable compositions (47), minimizing the variability in densities of *P. japonica* across different landscapes. The number of vineyards in our study did not enable us to perform a more complex analysis of multiple landscape characteristics, and the level of detail available to us from the Cropland Data Layer (NASS USDA 2017) did not allow us to calculate precise estimates of relative abundance of preferred host plants (19) and suitable oviposition sites (18), which could influence *P. japonica* populations. Future research with a greater number of study sites and a higher resolution of surrounding landscape composition may help elucidate finer scale landscape effects on *P. japonica* abundance.

Over the past century, *P. japonica* have gradually extended their range westward in the United States (38), and the larger abundance of adults observed in Eastern Wisconsin could be a result of an earlier establishment. A study on stink bug damage in Mid-Atlantic tomato fields observed a similar pattern with greater amounts of damage caused by the brown marmorated stink bug, *Halyomorpha halys* (Stål) (Hemiptera: Pentatomidae), in fields further east, closer to the invasion epicenter for this pest (11). Invasive species often have lag times where population densities in newly invaded areas are initially low and then later dramatically increase (48), therefore the population densities we observed could be influenced by *P. japonica*’s invasion history.

Alternatively, the geographical differences in population density could be attributed to other abiotic or biotic conditions varying across the state. The vineyards sampled in this study span a distance of over 200 km and are located in different broader ecological landscapes across Southern Wisconsin which vary in climate, soils, hydrology, and landcover (37), and vineyards located further east did generally have a slightly higher percent cropland in their surrounding landscapes. The variable conditions throughout the state could influence the establishment of *P. japonica* and their relative abundance across these locations (16, 38), but further work is needed to evaluate the specific landscape features that affect the abundance of *P. japonica*.

Previous research has shown the defoliation caused by *P. japonica* can have a negative effect on the growth and productivity of grape vines, and that the susceptibility to leaf injury and the subsequent impact on the vines is determined by the time of the season when leaf injury occurs (49), the age of the vine (50), and the specific cultivar (28). Here we showed that a combined effect of longitude, EIQ, and temperature best explained the estimated proportion of leaf injury within vineyards, with more leaf injury seen at vineyards that were further east, that had not used pesticides or used pesticides with lower environmental impact quotients, and had higher temperatures compared to vineyards that were further west, that had greater or more environmentally impactful pesticide usage, and experienced lower temperatures. As expected, vineyards with a higher EIQ generally had a lower estimated proportion of leaf injury. The most common active ingredient applied across the vineyards sampled was carbaryl (Sevin), which contributed to the EIQ scores and has been shown to reduce defoliation from *P. japonica* (16, 51). Past research has also shown *P. japonica* adults tend to have higher consumption rates at higher temperatures (30), consistent with our findings of greater leaf injury, and our collection of more beetles on vines at vineyards with higher mean temperatures.

Our results show that the weekly variability in *P. japonica* abundance is influenced by the previous week’s weather conditions of temperature and precipitation, with higher temperature and lower precipitation leading to higher adult abundance. Previous research has shown greater herbivory by *P. japonica* at higher temperatures in the laboratory (30) and that temperature and UV-radiation may encourage *P. japonica* adults to feed and aggregate on raspberry plants (52). Conversely, higher precipitation led to fewer *P. japonica* the following week on grapevines, consistent with historical observations of reduced flight activity and feeding of *P. japonica* during rain and dense cloud cover (53). A study that looked at variability between hourly abundance of *P. japonica* collected from traps with lures also found that flight activity and aggregation of *P. japonica* was sensitive to changes in weather conditions, capturing less beetles during hours with dense cloud cover and high winds (54).

As expected, our results showed a positive correlation between the abundance of *P. japonica* and estimated proportion of leaf injury and that more beetles and leaf injury was observed at the edges of vineyards compared to the interior of vineyards. This result is consistent with research in soybean and blueberry, which found a similar edge-biased spatial distribution for *P. japonica* (25, 26). One explanation for these results is that many of the adults found on grape vines moved in from the surrounding landscape, then started feeding on vines which immediately bordered the surrounding landscape. *Popillia japonica* respond to plant kairomones from feeding damage (55) and sex pheromones from female beetles, which can result in large aggregations of beetles feeding in concentrated locations (56). Alternatively, *P. japonica* adults which emerge from within the vineyards may start to distribute outwards and may stop at the vineyard edges in response to the distinct change in habitat (57), so these aggregations could result from beetles which had overwintered further within the vineyards. Edge effects, or edge-biased distributions, have been widely observed across many insect species, but theories explaining this phenomenon have not been extensively tested (58). Understanding the spatial distribution of pest insects is important as it can influence management practices, such as encouraging more targeted applications of insecticides (31). For *P. japonica* adults in vineyards, our results suggest that it may be more effective to focus management specifically near vineyard edges.

## Conclusions

Understanding the composition of the surrounding landscape can be important when assessing risk posed to crops by different pest insects. Our results showed that the abundance of *P. japonica* within vineyards was not significantly influenced by the amount of cropland in the surrounding landscape but rather was better explained by the amount of pasture, geographical location of the vineyard and temperature, and that leaf injury was influenced by geographical location of the vineyard, temperature, and pesticide usage. Additionally, we found weekly adult *P. japonica* abundance was greater when recent temperatures were higher and precipitation was lower. The edge-biased spatial distribution of *P. japonica* along linear transects within vineyards suggests that targeted management strategies to vineyard edges may be useful for reducing populations of adult beetles and their associated leaf injury.

## Data availability statement

The raw data supporting the conclusions of this article will be made available by the authors, without undue reservation.

## Author contributions

JH and CG designed the research. CG secured funding. JH collected and analyzed the data. JH and CG wrote the manuscript. All authors read and approved this manuscript.

## Funding

This study was funded by the UW-Madison Department of Entomology Lilian and Alex Feir Fellowship and a USDA North Central Region Sustainable Agriculture Research and Education program grant project number GNC 17-239.

## Acknowledgments

We give special thanks to all the participating growers for access to their vineyards to conduct this study. We also thank Sarah Woody, Michaela Taddeini, and Cybil Biehlmann for helping with sample collection and processing.

## Conflict of interest

The authors declare that the research was conducted in the absence of any commercial or financial relationships that could be construed as a potential conflict of interest.

## Publisher’s note

All claims expressed in this article are solely those of the authors and do not necessarily represent those of their affiliated organizations, or those of the publisher, the editors and the reviewers. Any product that may be evaluated in this article, or claim that may be made by its manufacturer, is not guaranteed or endorsed by the publisher.

## Supplementary material

SupplementaL Table 1 This table describes various characteristics of the vineyards in our study, as well as a summary of collected data used in our analysis, the table includes the following columns: 1) A vineyard identification label for each vineyard V01-V20 (VID); 2) Latitude of the vineyard in decimal degrees (Lat); 3) Longitude of the vineyard in decimal degrees (Long); 4) Area of the vineyard in hectares [Area (ha)]; 5) Length of diagonal transect established at the vineyard [Transect (m)]; 6) Proportion of surrounding 1.5km landscape defined as cropland (Cropland); 7) Proportion of surrounding 1.5km landscape defined as woodland (Woodland); 8) Proportion of surrounding 1.5km landscape defined as Non-Pasture Grassland (Non-Pasture Grassland); 9) Proportion of surrounding 1.5km landscape defined as Pasture (Pasture); 10) Average precipitation (mm) from June-September for 2017 [2017 Precip (mm)]; 11) Average temperature(°C) from June-September for 2017 [2017 Temp (°C)]; 12) Average precipitation (mm) from June-September for 2018 [2018 Precip (mm)]; 13) Average temperature(°C) from June-September for 2018 [2018 Temp (°C)]; 14) Average precipitation (mm) from June-September for 2017 and 2018 [2017,2018 Precip (mm)]; 15) Average temperature(°C) from June-September for 2017 and 2018 [2017,2018 temperature(°C)]; 16) Environmental Impact Quotient Estimates for June-September 2017 (2017 EIQ); 17) Environmental Impact Quotient Estimates for June-September 2018 (2018 EIQ); Average Environmental Impact Quotient Estimates for June-September 2017 and 2018 (2017,2018 EIQ); 18) Number of collected *P. japonica* adults during sampling in 2017 (2017 P*. japonica*); 19) Number of collected *P. japonica* adults during sampling in 2018 (2018 P*. japonica*); 20) Average number of collected *P. japonica* adults during sampling in 2017 and 2018 (2017,2018 *P. japonica*); 21) Average estimated leaf injury in 2017 (2017 leaf injury); 22) Average estimated leaf injury in 2018 (2018 leaf injury); 23) Average estimated leaf injury across 2017 and 2018 (2017,2018 leaf injury).

SupplementaL Table 2 Reclassification table of USDA NASS land cover classes.

SupplementaL Table 3 The AIC, ΔAIC (difference between AIC of the model and AIC of the selected model), Adjusted R square, and p-values of the 37 simple or multiple regression models produced to examine variables to explain the variation in average adult P. japonica abundance (log transformed) across vineyards. Variables included in the model selection included: 1) proportion of the surrounding landscape covered in cropland (woodland); 2) averaged daily precipitation (cm) from June-September of 2017 and 2018 at the vineyard (precip); 3) averaged daily temperature (°C) from June-September of 2017 and 2018 at the vineyard (temp); 4) Environmental Impact quotient score for the vineyard averaged across 2017 and 2018 (EIQ); and 5) longitude value of the center of each vineyard (long). No more than three variables were included in single model. The selected model with the lowest AICc shown in bold.

SupplementaL Table 4 The AICc, ΔAICc (difference between AICc of the model and AICc of the selected model), Adjusted R square, and p-values of the 37 simple and multiple linear regression models with interaction effects produced to examine variables to explain the variation in average estimated proportion of leaf injury across vineyards. Variables included in the model selection included: 1) proportion of the surrounding landscape covered in cropland (woodland); 2) averaged daily precipitation (cm) from June-September of 2017 and 2018 at the vineyard (precip); 3) averaged daily temperature (°C) from June-September of 2017 and 2018 at the vineyard (temp); 4) Environmental Impact quotient score for the vineyard averaged across 2017 and 2018 (EIQ); and 5) longitude value of the center of each vineyard (long). No more than three variables were included in single model. The selected model with the lowest AICc shown in bold.

SupplementaL Table 5 The following table represents models we built exploring weekly variability in adult P.japonica (log transformed to meet assumptions of normality) across 20 vineyards. The variables included in models were 1) Week (the week we sampled, 1st week for the year; 2) Year (year of sampling, 2017 or 2018); 3) Vin (vineyard sampled V01-V20); 4) Precip (average precipitation at the vineyard sampled for the previous 7 days); and 5) average temperature at the vineyard sampled for the previous 7 days. The AIC, ΔAIC (difference between AIC of the model and AIC of the selected model), Adjusted R square, and p-values are shown for all models.

SupplementaL Table 6 Parameter estimates ± SE of selected model (R2=0.51, p<0.001) explaining variation in the average weekly abundance of average P. japonica adults per vineyard (transformed by log base 10). Variables included in the selected model were: 1) Week (quadratic term added to fit the data); 2)Year ( 2017 or 2018); 3) Vineyard (V02-V20) (vineyards were considered categorical in this model, so coefficients for vineyards V02-V20 are shown relative to V01); 4) average weekly temperature (°C) representing an average of 7 days of daily temperature consisting of the 6 days prior to when sampling occurred along with the day of sampling itself (Temp) and ; 5) average weekly precipitation (mm) representing an average of 7 days of daily temperature consisting of the 6 days prior to when sampling occurred along with the day of sampling itself (Precip).

SupplementaL Table 7 This table includes all data collected from vineyards in our study for and includes the following columns: 1) Day of the year (Day); 2) Month of the year (Month); 3) Year of the study, 2017 or 2018 (Year); 4) Time of day when sampling occurred (Time); Week of the study in the given year (Week); Vineyard Identification label for the 20 vineyards, V01-V20 (VID); Point Identification for sampling points along the transect P1 = Edge of vineyard, P2 = halfway from edge to center, P2 = center of vineyard (PID); Indicated which of the 6 vines associated with each sampling point was used for leaf injury estimates, vines sequentially numbered along the row of plants starting at the beginning of the sampling point moving further into the vineyard (Vine Number); 9) Indicates where on a grape plant sampling took place from the perspective of the collector standing in front of the plant within the vineyard row, RC = Right cordons, or shoots on the right side of the plant, LC = Left Cordons, or shoots on the left side of the plant, EV = Entire Vine, or collecting from both left and right side of the plant in order to count up to 40 leaves (Vine Cordon); 10) Number of leaves counted, counts less than 40 indicate a limited number of leaves on the plant of adequate size to assess (Leaves Counted); 11) Out of all of the leaves counted, the number that were visually estimated to have at least 10% leaf area loss (Leaved Injured);12) The proportion of damaged leaves out of all leaves counted (Proportion Leaf Injury); 13) The number of *P. japonica* adults collected (*P. japonica*); 14) The number of *P. japonica* female adults collected (*P. japonica* Females); 15) The number of *P. japonica* male adults collected (*P. japonica* Males); 16) Average daily precipitation (mm) [Precip (mm)]; 17) Average daily temperature [Temp (°C)]; 18) Average weekly precipitation for the day of sampling and previous 6 days [Weekly Precip (mm)]; 19) Average weekly temperature for the day of sampling and previous 6 days [Weekly Temp (°C)].
