# Supplementary material for: Effect of surrounding landscape on Popillia japonica abundance and their spatial pattern within Wisconsin vineyards
Source: Front Insect Sci. 2022 Oct 27;2:961437. doi: 10.3389/finsc.2022.961437 (PMC10926536; doi:10.3389/finsc.2022.961437)
Supplement: SupplementaL Table 2 — Reclassification table of USDA NASS land cover classes. [file Table_2.docx]

**Supplemental Table 2.** Reclassification table of USDA NASS land cover classes

| **New Class Name** | **Original USDA NASS Cropland Data Layer Class Names (2017)** |
| --- | --- |
| **Cropland** | Alfalfa, Barley, Corn, Dry Beans, Herbs, Oats, Onions, Potatoes, Rye, Soybean, Spring Wheat, Sunflower, Winter Wheat, |
| **Woodland** | Deciduous Forests, Evergreen Forest, Mixed Forest |
| **Non-pasture Grassland** | Barren, Clover/Wildflowers, Fallow/Idle Cropland, Other Hay/Non Alfalfa, Herbaceous Wetland, Shrubland |
| **Pasture** | Grass/Pasture |
