# Supplementary material for: Effect of surrounding landscape on Popillia japonica abundance and their spatial pattern within Wisconsin vineyards
Source: Front Insect Sci. 2022 Oct 27;2:961437. doi: 10.3389/finsc.2022.961437 (PMC10926536; doi:10.3389/finsc.2022.961437)
Supplement: SupplementaL Table 3 — The AIC, ΔAIC (difference between AIC of the model and AIC of the selected model), Adjusted R square, and p-values of the 37 simple or multiple regression models produced to examine variables to explain the variation in average adult P. japonica abundance (log transformed) across vineyards. Variables included in the model selection included: 1) proportion of the surrounding landscape covered in cropland (woodland); 2) averaged daily precipitation (cm) from June-September of 2017 and 2018 at the vineyard (precip); 3) averaged daily temperature (°C) from June-September of 2017 and 2018 at the vineyard (temp); 4) Environmental Impact quotient score for the vineyard averaged across 2017 and 2018 (EIQ); and 5) longitude value of the center of each vineyard (long). No more than three variables were included in single model. The selected model with the lowest AICc shown in bold. [file Table_3.docx]

**Supplemental Table 3.** The AIC, ΔAIC (difference between AIC of the model and AIC of the selected model), Adjusted R square, and p-values of the 37 simple or multiple regression models produced to examine variables to explain the variation in average adult *P. japonica* abundance (log transformed) across vineyards. Variables included in the model selection included: 1) proportion of the surrounding landscape covered in cropland (woodland); 2) averaged daily precipitation (cm) from June-September of 2017 and 2018 at the vineyard (precip); 3) averaged daily temperature (°C) from June-September of 2017 and 2018 at the vineyard (temp); 4) Environmental Impact quotient score for the vineyard averaged across 2017 and 2018 (EIQ); and 5) longitude value of the center of each vineyard (long). No more than three variables were included in single model. The selected model with the lowest AICc shown in bold.

| **Model** | **Variables and interaction effects included** | **AICc** | **ΔAICc** | **Adjusted R-square** | **p value** |
| --- | --- | --- | --- | --- | --- |
| **1** | cropland | 60.24 | 23.02 | 0.02 | 0.27 |
| **2** | precip | 60.29 | 23.07 | 0.01 | 0.28 |
| **3** | eiq | 61.40 | 24.18 | -0.04 | 0.64 |
| **4** | temp | 54.67 | 17.45 | 0.26 | 0.01 |
| **5** | long | 51.03 | 13.81 | 0.38 | <0.01 |
| **6** | cropland + precip | 62.31 | 25.09 | 0.01 | 0.35 |
| **7** | cropland + eiq | 63.37 | 26.15 | -0.04 | 0.54 |
| **8** | cropland + eiq + cropland:eiq | 64.88 | 27.66 | 0.01 | 0.40 |
| **9** | cropland + temp | 56.31 | 19.09 | 0.27 | 0.03 |
| **10** | precip + eiq | 63.45 | 26.23 | -0.04 | 0.56 |
| **11** | precip + temp | 55.28 | 18.06 | 0.31 | 0.02 |
| **12** | precip + long | 53.36 | 16.14 | 0.37 | 0.01 |
| **13** | eiq + temp | 57.51 | 20.29 | 0.22 | 0.04 |
| **14** | eiq + long | 54.12 | 17.2 | 0.35 | 0.01 |
| **15** | temp + long | 39.69 | 2.47 | 0.68 | <0.01 |
| **16** | cropland + precip + eiq | 65.88 | 28.66 | -0.05 | 0.55 |
| **17** | cropland + precip + temp | 57.70 | 20.48 | 0.31 | 0.03 |
| **18** | cropland + eiq + temp | 59.85 | 22.63 | 0.23 | 0.07 |
| **19** | cropland + long + temp | 42.76 | 5.54 | 0.67 | <0.01 |
| **20** | precip + eiq + temp | 58.90 | 20.78 | 0.26 | 0.05 |
| **21** | precip + eiq + long | 56.51 | 19.29 | 0.35 | 0.02 |
| **22** | long + temp + eiq | 43.11 | 5.89 | 0.67 | <0.01 |
| **23** | pasture | 61.57 | 24.35 | 0.05 | 0.79 |
| **24** | pasture+precip | 63.35 | 26.13 | 0.04 | 0.54 |
| **25** | pasture+eiq | 64.41 | 27.19 | 0.09 | 0.84 |
| **26** | pasture+temp | 56.92 | 19.70 | 0.25 | 0.03 |
| **27** | pasture+long | 42.61 | 4.88 | 0.63 | <0.01 |
| **28** | pasture+cropland | 63.19 | 25.97 | 0.03 | 0.50 |
| **29** | pasture+cropland+pasture:cropland | 66.52 | 29.30 | 0.08 | 0.66 |
| **30** | pasture+cropland+precip | 65.68 | 28.46 | 0.03 | 0.52 |
| **31** | pasture+cropland+eiq | 66.73 | 29.51 | 0.09 | 0.70 |
| **32** | pasture+cropland+temp | 59.33 | 22.11 | 0.25 | 0.06 |
| **33** | pasture+eiq+pasture:eiq | 67.89 | 30.67 | 0.16 | 0.93 |
| **34** | pasture+precip+eiq | 66.95 | 29.73 | 0.10 | 0.75 |
| **35** | pasture+precip+temp | 57.87 | 20.65 | 0.30 | 0.03 |
| **36** | pasture+eiq+temp | 60.40 | 23.18 | 0.20 | 0.08 |
| **37** | **pasture+long+temp** | **37.22** | **0.00** | **0.75** | **<0.01** |
