# Supplementary material for: Effect of surrounding landscape on Popillia japonica abundance and their spatial pattern within Wisconsin vineyards
Source: Front Insect Sci. 2022 Oct 27;2:961437. doi: 10.3389/finsc.2022.961437 (PMC10926536; doi:10.3389/finsc.2022.961437)
Supplement: SupplementaL Table 5 — The following table represents models we built exploring weekly variability in adult P.japonica (log transformed to meet assumptions of normality) across 20 vineyards. The variables included in models were 1) Week (the week we sampled, 1st week for the year; 2) Year (year of sampling, 2017 or 2018); 3) Vin (vineyard sampled V01-V20); 4) Precip (average precipitation at the vineyard sampled for the previous 7 days); and 5) average temperature at the vineyard sampled for the previous 7 days. The AIC, ΔAIC (difference between AIC of the model and AIC of the selected model), Adjusted R square, and p-values are shown for all models. [file Table_5.docx]

**Supplemental Table 5.**

The following table represents models we built exploring weekly variability in adult *P.japonica* (log transformed to meet assumptions of normality) across 20 vineyards. The variables included in models were 1) Week (the week we sampled, 1^st^ week for the year; 2)Year (year of sampling, 2017 or 2018); 3) Vin (vineyard sampled V01-V20); 4) Precip (average precipitation at the vineyard sampled for the previous 7 days); and 5) average temperature at the vineyard sampled for the previous 7 days. The AIC, ΔAIC (difference between AIC of the model and AIC of the selected model), Adjusted R square, and p-values are shown for all models.

| **Model** | **Variables and interaction effects included** | **AIC** | **ΔAIC** | **Adjusted R-square** | **p value** |
| --- | --- | --- | --- | --- | --- |
| **1** | week^2^+week+year+vin | 1957.46 | 8.24 | 0.50 | <0.001 |
| **2** | week^2^+week+year+vin+precip | 1955.24 | 6.02 | 0.50 | <0.001 |
| **3** | week^2^+week+year+vin+temp | 1952.71 | 3.49 | 0.51 | <0.001 |
| **4** | **week^2^+week+year+vin+precip+temp** | **1949.22** | **0.00** | **0.51** | **<0.001** |
| **5** | week^2^+week+year+vin+precip+temp+precip:temp | 1950.44 | 1.22 | 0.51 | <0.001 |
