# Supplementary material for: Effect of surrounding landscape on Popillia japonica abundance and their spatial pattern within Wisconsin vineyards
Source: Front Insect Sci. 2022 Oct 27;2:961437. doi: 10.3389/finsc.2022.961437 (PMC10926536; doi:10.3389/finsc.2022.961437)
Supplement: SupplementaL Table 6 — Parameter estimates ± SE of selected model (R2=0.51, p<0.001) explaining variation in the average weekly abundance of average P. japonica adults per vineyard (transformed by log base 10). Variables included in the selected model were: 1) Week (quadratic term added to fit the data); 2)Year ( 2017 or 2018); 3) Vineyard (V02-V20) (vineyards were considered categorical in this model, so coefficients for vineyards V02-V20 are shown relative to V01); 4) average weekly temperature (°C) representing an average of 7 days of daily temperature consisting of the 6 days prior to when sampling occurred along with the day of sampling itself (Temp) and ; 5) average weekly precipitation (mm) representing an average of 7 days of daily temperature consisting of the 6 days prior to when sampling occurred along with the day of sampling itself (Precip). [file Table_6.docx]

**Supplemental Table 6.** Parameter estimates ± SE of selected model (R^2^=0.51, *p*<0.001) explaining variation in the average weekly abundance of average *P. japonica* adults per vineyard (transformed by log base 10). Variables included in the selected model were: 1) Week (quadratic term added to fit the data); 2)Year ( 2017 or 2018); 3) Vineyard (V02-V20) (vineyards were considered categorical in this model, so coefficients for vineyards V02-V20 are shown relative to V01); 4) average weekly temperature (°C) representing an average of 7 days of daily temperature consisting of the 6 days prior to when sampling occurred along with the day of sampling itself (Temp) and ; 5) average weekly precipitation (mm) representing an average of 7 days of daily temperature consisting of the 6 days prior to when sampling occurred along with the day of sampling itself (Precip)

| **Coefficients** | **Parameter Estimate ± SE** | **t value** | **Pr>(\|t\|)** |
| --- | --- | --- | --- |
| **Intercept** | 286.25±213.42 | 1.34 | 0.18 |
| **Week^2^** | -0.04±0.003 | -15.67 | <0.0001 |
| **Week** | 0.76±0.05 | 16.18 | <0.0001 |
| **Year** | -0.14±0.11 | -1.35 | 0.18 |
| **V02** | -0.51 ± 0.30 | -1.66 | 0.10 |
| **V03** | -1.66±0.31 | -5.31 | <0.0001 |
| **V04** | -1.04±0.31 | -3.39 | <0.001 |
| **V05** | -1.85±0.31 | -5.89 | <0.0001 |
| **V06** | -1.86±0.31 | -5.95 | <0.0001 |
| **V07** | 0.60±0.31 | 1.96 | 0.05 |
| **V08** | -0.08±0.30 | -0.26 | 0.80 |
| **V09** | 0.39±0.31 | 1.26 | 0.21 |
| **V10** | -0.57±0.31 | -1.84 | 0.07 |
| **V11** | 0.54±0.30 | 1.78 | 0.07 |
| **V12** | 0.31±0.30 | 0.99 | 0.32 |
| **V13** | 1.13±0.31 | 3.70 | <0.001 |
| **V14** | 1.29±0.30 | 4.28 | <0.0001 |
| **V15** | -0.38±0.31 | -1.20 | 0.23 |
| **V16** | 0.99±0.30 | 3.26 | <0.01 |
| **V17** | -0.52±0.31 | -1.63 | 0.10 |
| **V18** | -1.86±0.31 | -5.94 | <0.001 |
| **V19** | -0.21±0.30 | -0.69 | <0.0001 |
| **V20** | 0.06±0.30 | 0.19 | 0.85 |
| **Temp** | 0.07±0.02 | 2.78 | <0.01 |
| **Precip** | 0.02±0.008 | -2.30 | 0.02 |
